# Accumulation and processing of a recombinant protein designed as a cleavable fusion to the endogenous Rubisco LSU protein in *Chlamydomonas *chloroplast

**DOI:** 10.1186/1472-6750-9-26

**Published:** 2009-03-26

**Authors:** Machiko Muto, Ryan E Henry, Stephen P Mayfield

**Affiliations:** 1 The Department of Cell Biology and The Skaggs Institute for Chemical Biology, The Scripps Research Institute, 10550 N. Pines Rd. La Jolla, CA 92037, USA; 2 Current address : The Department of Molecular, Cellular and Developmental Biology, The University of Colorado, Boulder, 347 UCB Boulder, CO 80309, USA

## Abstract

**Background:**

Expression of recombinant proteins in green algal chloroplast holds substantial promise as a platform for the production of human therapeutic proteins. A number of proteins have been expressed in the chloroplast of *Chlamydomonas reinhardtii*, including complex mammalian proteins, but many of these proteins accumulate to significantly lower levels than do endogenous chloroplast proteins. We examined if recombinant protein accumulation could be enhanced by genetically fusing the recombinant reporter protein, luciferase, to the carboxy-terminal end of an abundant endogenous protein, the large subunit of ribulose bisphosphate carboxylase (Rubisco LSU). Additionally, as recombinant proteins fused to endogenous proteins are of little clinical or commercial value, we explored the possibility of engineering our recombinant protein to be cleavable from the endogenous protein *in vivo*. This strategy would obviate the need for further *in vitro *processing steps in order to produce the desired recombinant protein. To achieve this, a native protein-processing site from preferredoxin (*preFd*) was placed between the Rubisco LSU and luciferase coding regions in the fusion protein construct.

**Results:**

The luciferase from the fusion protein accumulated to significantly higher levels than luciferase expressed alone. By eliminating the endogenous Rubisco large subunit gene (*rbcL*), we achieved a further increase in luciferase accumulation with respect to luciferase expression in the WT background. Importantly, near-wild type levels of functional Rubisco holoenzyme were generated following the proteolytic removal of the fused luciferase, while luciferase activity for the fusion protein was almost ~33 times greater than luciferase expressed alone. These data demonstrate the utility of using fusion proteins to enhance recombinant protein accumulation in algal chloroplasts, and also show that engineered proteolytic processing sites can be used to liberate the exogenous protein from the endogenous fusion partner, allowing for the purification of the intended mature protein.

**Conclusion:**

These results demonstrate the utility of fusion proteins in algal chloroplast as a method to increase accumulation of recombinant proteins that are difficult to express. Since Rubisco is ubiquitous to land plants and green algae, this strategy may also be applied to higher plant transgenic expression systems.

## Background

The use of transgenic plant expression systems has recently become of great interest to biotechnology, as plants represent a potentially robust and economic platform for recombinant protein production [1]. The use of plants and green algal chloroplasts for transgenic protein expression has some practical advantages compared to that of nuclear expression. These advantages include the absence of gene silencing, the ability to precisely target the gene of interest to specific regions of the chloroplast genome by homologous recombination, the potential for robust expression of heterologous proteins, rapid generation of stable transgenic lines, simple promoter and expression elements, and limited post-translational modifications to the recombinant protein [2-4]. Furthermore, large-scale algal production can be undertaken in contained facilities where the probability of environmental spread of transgenes or product contamination is greatly reduced compared to terrestrial plants grown in open fields [3]. Thus far, chloroplast transformation has been routinely employed only in tobacco and *Chlamydomonas*, although chloroplast transformation has been reported for several other plant species [5].

Expression of recombinant proteins in the *Chlamydomonas *chloroplast is now well established [3], although expression levels vary considerably between proteins. The *Chlamydomonas *chloroplast occupies a large proportion of the cell volume (~60%), with sufficient capacity for significant exogenous protein accumulation [6,7]. Moreover, methods for transforming the *Chlamydomonas *chloroplast genome are relatively simple [8-10], and chloroplast transformants can be selected through co-transformation with DNA conferring resistance to antibiotics [11-13] or through phototrophic rescue [10].

In order to achieve high levels of recombinant protein expression in the *C. reinhardtii *chloroplast, codon-optimized reporter genes were developed [14,15] and used to examine a variety of promoter and translational elements [16]. Using this strategy, GFP accumulation up to 0.5% of total soluble protein (TSP) was achieved in transgenic chloroplasts [15,16]. Although these expression levels were sufficient for reporter gene measurements, overall this level of protein expression is low relative to other protein expression systems. A synthetic luciferase gene, also optimized to suit the *C. reinhardtii *chloroplast codon bias (*luxCt*), was used to assay heterologous gene expression under a variety of growth conditions [14]. Using the *luxCt *gene, growth conditions for optimal gene expression in chloroplast were successfully determined, but protein accumulation of the LUXCt protein was again still modest, at less than 0.1% TSP [14].

The *rbcL *gene, which encodes the ribulose bisphosphate carboxylase/oxygenase large subunit (Rubisco LSU), may present a unique strategic target for improving transgenic protein accumulation in the chloroplast. The Rubisco LSU promoter and translation elements have been used to drive expression of exogenous reporter genes in *C. reinhardtii *[17], but expression using these elements alone did not appear to be improved over other chloroplast promoters and translation elements [16]. Kuroda and colleagues made fusions of the first 14 N-terminal amino acids of either the Rubisco LSU or ATP synthase β-subunit (encoded by the *atpB *gene) to a neomycin phosphotransferase (NPTII) reporter enzyme and showed that silent mutations made to the first 14 codons of the *rbcL-NPTII *fusion caused a 35-fold decrease in *NPTII *accumulation, whereas similar mutations to the *atpB-NPTII *fusion resulted in a less than 2-fold decrease in reporter protein accumulation [18]. As the introduction of silent mutations did not result in the creation of rare codons, decreased reporter protein accumulation was attributed to effects of mRNA sequences downstream of the translation initiation codon on protein accumulation. Kasai *et al*. have also demonstrated the utility of using the *rbcL *coding region to increase expression of recombinant proteins [19]. From such studies, it seems likely that mRNA sequences downstream of the promoter/5' untranslated region (UTR) contribute to the efficient translation of some plastid mRNAs, although the precise role of these elements remains elusive. Furthermore, it is tempting to speculate that genetic fusions of efficiently-translated, highly-abundant chloroplast proteins to an exogenous protein of interest may represent an effective strategy for high-level transgene expression. Since Rubisco is commonly noted to be the most abundant protein in photosynthetic organisms, recombinant protein fusions to the chloroplast-encoded Rubisco LSU may potentially be used to enhance the accumulation of poorly-expressed recombinant proteins in the chloroplast. Fusion to Rubisco LSU has already been reported for a small peptide in tobacco [20]. Although fusion proteins have been used successfully in many protein expression systems to improve protein accumulation, this strategy has not been attempted in the *Chlamydomonas *chloroplasts.

This study provides the first example of genetically manipulating an algal photosynthetic protein to increase recombinant protein accumulation. By introducing an *rbcL-luxCt *fusion gene we could show a substantial increase in luciferase accumulation compared to expression of the *luxCt *gene alone. Insertion of a proteolytic processing site between the Rubisco LSU and LUXCt coding regions in the fusion protein construct allowed for the generation of separate Rubisco LSU and LUXCt proteins upon processing by an endogenous chloroplast protease. We also demonstrate that Rubisco LSU protein derived solely from the fusion protein construct is completely functional, as evidenced by normal photosynthesis in transformants where the endogenous *rbcL *gene has been eliminated. Since Rubisco is ubiquitous to land plants and green algae, this strategy may be potentially applied to other plant transgenic expression systems.

## Results

### Construction of the *rbcL-luxCt *fusion and *rbcL-aphA6 *replacement genes in the *C. reinhardtii *chloroplast

**Figure 1:**
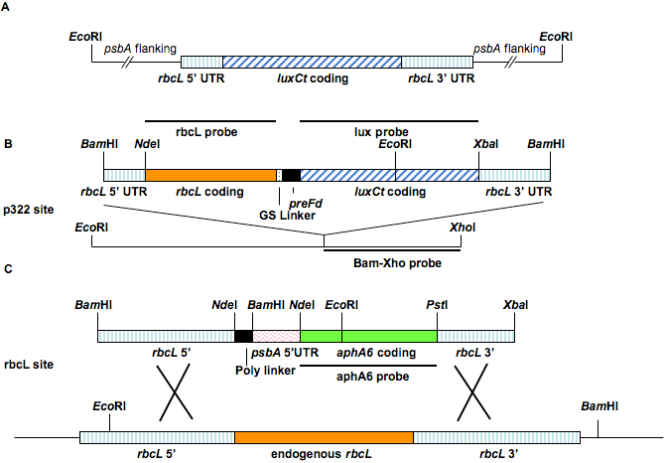
**Maps of *luxCt*, *rbcL-luxCt/rbcL*^*wt*^, and *rbcL-luxCt/rbcL*^- ^constructs for expression in *C. reinhardtii *chloroplasts**. **A **Schematic diagram of the replaced region, including relevant restriction sites. Homologous regions used for recombination between the insertion plasmid and the *C. reinhardtii *chloroplast genome are shown as flanking genome regions, and insert *psbA *sites in the chloroplast genome. **B **Map of the vector targeting the p322 inverted-repeat within the chloroplast. Relevant restriction sites delineate the *rbcL *5' UTR (*Bam*HI-*Nde*I), *rbcL *and *luxCt *coding regions, linker and preferredoxin transit peptide (*preFd*) regions, and the *rbcL *3' UTR (*Xba*I-*Bam*HI). Map showing the homologous region between the p322 plasmid and the *C. reinhardtii *chloroplast genome into which the chimeric *rbcL-luxCt *fusion gene was integrated. *C. reinhardtii *chloroplast DNA is depicted as the *Eco*RI to *Xho*I fragment of 5.7-kb located in the inverted repeat region of the chloroplast genome. **C **Transgene replacement of the endogenous *rbcL *gene through *rbcL *5' and 3' homologous recombination (crossed lines) thereby knocking-out the original *rbcL *coding region with the *aphA6 *kanamycin-resistance gene. The thick black lines indicate regions corresponding to the probes used in the Southern and Northern blot analysis.

To achieve high levels of heterologous protein expression in the *C. reinhardtii *chloroplast, chimeric genes were codon-optimized to reflect abundantly-expressed genes of the *Chlamydomonas *chloroplast [14,15]. For luciferase expression in the chloroplast, we first constructed the expression cassette shown in Figure 1A, in which the *Chlamydomonas *chloroplast luciferase reporter gene *luxCt *[14], based on the bacterial luciferase AB gene of *V. harveyi *[21] was ligated downstream of the *rbcL *promoter and 5' UTR [16]. We had previously constructed the vector as an internal standard for replacing the endogenous *psbA *gene, thereby generating a non-photosynthetic strain, and showed that expression of the *luxCt *reporter using this *rbcL *promoter and 5' UTR achieved recombinant protein accumulation to about 0.05% of total protein [7]. This construct served as the standard for comparison with the fusion proteins described below.

To determine if we could achieve higher levels of recombinant protein accumulation by fusing a recombinant protein with a highly expressed endogenous protein, we assembled the chimeric gene construct shown in Figure 1B. In this construct the *luxCt *gene was fused in frame and downstream of the *rbcL *coding sequence with a 14 amino acid linker (GS linker; GGGGSSGGGGGGSS) and a preferredoxin (*preFd*) enzymatic cleavage site between the two genes [22]. The preFd enzymatic cleavage site was chosen as a protease site that should be cleaved by the endogenous preFd-processing enzyme normally found in the chloroplast. This cleavage site provided the potential to generate separate LUXCt and Rubisco LSU proteins following translation by site-specific cleavage of the fusion protein at amino acid MAMAMRSTFAARVGAKPAVRGARPASRMSCMA to generate two separate proteins. The linker and processing sites were assembled from oligonucleotides using the PCR-based method described previously [23]. PCR products were cloned into *Escherichia coli *plasmids and ligated to generate the final fusion construct. An *Nde*I site was placed at the *rbcL *initiation codon, and an *Xba*I site was placed downstream of the *luxCt *stop codon, to facilitate subsequent cloning steps. The *rbcL-luxCt *fusion coding sequence was ligated downstream of the *rbcL *promoter and 5' UTR, and upstream of the *rbcL *3' UTR (Figure 1B). The chimeric gene was then ligated into the chloroplast transformation plasmid p322 [14,15] at the unique *Bam*HI site to create plasmid p322-*rbcL-luxCt*. This construct was integrated into the p322 integration site using a spectinomycin resistance selectable marker gene as previously described [15].

To determine whether the Rubisco LSU protein was still functional (as either the monomeric form generated by preFd cleavage of the Rubisco LSU-LUXCt fusion protein or as a fusion to the LUXCt protein), we constructed a knock-out vector in which the endogenous *rbcL *gene in strains expressing the *rbcL-luxCt *fusion could be deleted by replacement of the endogenous *rbcL *gene with the kanamycin resistance selectable marker *aphA6*, as shown in Figure 1C[12]. A codon-optimized *aphA6 *gene, driven by the *psbA *promoter and 5' UTR, was ligated in place of the coding region of the *rbcL *gene, but leaving 800 bp of genomic sequence on the 5' end of the *rbcL *locus and 450 bp on the 3' end of the *rbcL *locus. These sequences were retained to allow sufficient homology for integration of the *aphA6 *gene as a direct replacement of the *rbcL *coding region (Figure 1C). Since the Rubisco LSU-LUXCt fusion protein provided the only source of Rubisco LSU in this strain, we were able to determine whether *rbcL-luxCt *was processed to restore Rubisco function by growing these strains photoautotrophically.

### Model of the Rubisco LSU-luciferase fusion protein

**Figure 2:**
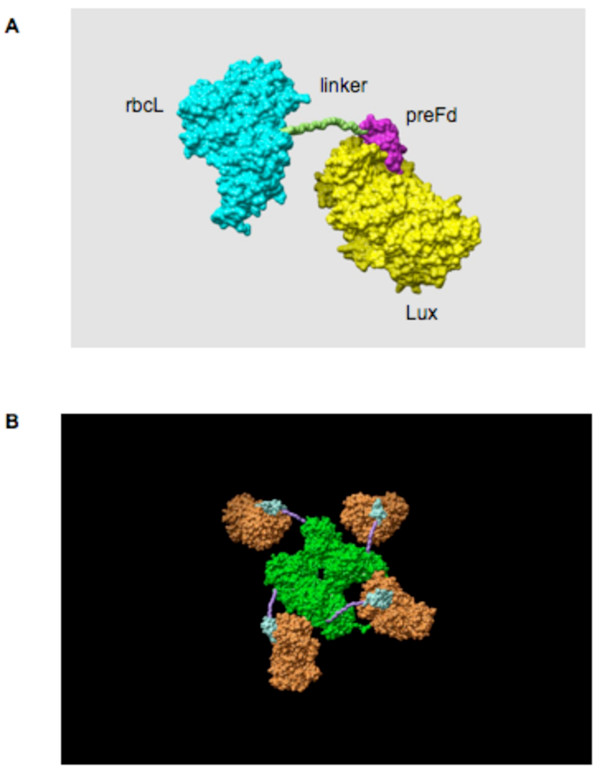
**Model of Rubisco LSU-LUXCt fusion protein structure**. **A **Predicted structure of a Rubisco LSU-LUXCt monomer. The GS linker (green) and preferredoxin protease site (preFd; purple) bridge the Rubisco LSU (blue) and a LUXCt (yellow). **B **Tetramer structure of the Rubisco LSU-LUX fusion.

The structure of the Rubisco LSU-LUXCt fusion protein was modeled (Figure 2) using the CHIMERA program (University of California San Francisco) and the data from the Protein Data Bank (PDB) public database. The structure of a Rubisco LSU-LUXCt fusion protein monomer (Figure 2A) and tetramer (Figure 2B) were predicted. The primary and tertiary structures of Rubisco LSUs were found to be highly similar between *C. reinhardtii *and other species [24]. The GS linker and preFd protease site [25] that bridge the Rubisco LSU [24] and the LUXCt protein are estimates based on their relative size compared to LUXCt and Rubisco LSU proteins [26,27]. As shown in Figure 2, cleavage of the preFd protease site should leave the Rubisco LSU with a short GS linker peptide on the carboxy terminus external to the protein, which is predicted to have little impact on Rubisco holoenzyme assembly or enzymatic function.

### Transformation and integration of *rbcL-luxCt *and *rbcL-aphA6 *genes into *C. reinhardtii *chloroplasts

Wild-type (WT) *C. reinhardtii *cells were transformed with the p322-*rbcL-luxCt *plasmid and the selectable marker plasmid p228, conferring resistance to spectinomycin [13,28]. Primary transformants were screened by Southern blot analysis, and positive transformants were taken through additional rounds of selection to isolate homoplasmic lines in which all copies of the chloroplast genome contained the integrated *luxCt *gene. A homoplasmic *rbcL-luxCt *transformant, still containing the wild type copy of the *rbcL *gene, *rbcL-luxCt/rbcL*^*wt*^, was selected for further experiments.

**Figure 3:**
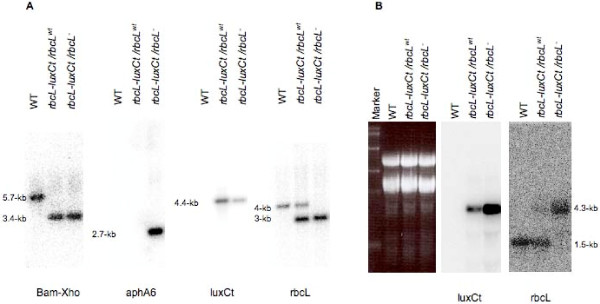
**Southern and Northern blot analysis of *rbcL-luxCt *fusion (*rbcL-luxCt/rbcL*^*wt*^) and combined *rbcL-luxCt *fusion endogenous *rbcL *knock-out (*rbcL-luxCt/rbcL*^-^) strains**. Lane 1, untransformed wild type (WT); lane 2, *rbcL-luxCt *fusion transformant *rbcL-luxCt/rbcL*^*wt*^; lane 3, combined *rbcL-luxCt *fusion/*rbcL *knock-out transformant (*rbcL-luxCt/rbcL*^-^). **A ***C. reinhardtii *DNA was digested with *Eco*RI and *Xho*I, and hybridized with the Bam-Xho probe (left panel), *Bam*HI for aphA6 (second panel from the left) or lux probes (third panel from left), and *Eco*RI and *Bam*HI for rbcL probes (right panel), respectively. **B **Detection of *luxCt *and *rbcL *mRNA expression in transgenic *C. reinhardtii *transformants. Total RNA isolated from WT, *rbcL-luxCt/rbcL*^*wt*^, and *rbcL-luxCt/rbcL*^- ^was separated on denaturing agarose gels (left panel) and blotted onto nylon membrane. The membranes were hybridized with *luxCt *(middle panel) or *rbcL *(right panel) cDNA probes.

The *rbcL-luxCt/rbcL*^*wt *^fusion line was subsequently transformed with the *rbcL-aphA6 *plasmid to generate the *rbcL-luxCt/rbcL*^- ^line, and transformants were selected on kanamycin plates. Integration of the recombinant genes and replacement of the endogenous *rbcL *gene were confirmed by Southern blot analysis (Figure 3A). The *rbcL-luxCt *and *rbcL-aphA6 *constructs with relevant restriction sites are indicated in Figure 1. Correct integration of the plasmid p322-*rbcL-luxCt *into the chloroplast genome was ascertained using probes to both the *luxCt *coding region and the *Bam*HI-*Xho*I fragment of plasmid p322. Replacement of the endogenous *rbcL *gene was confirmed using probes to both the *aphA6 *and *rbcL *coding regions (Figure 3A). Genomic DNA from WT, *rbcL-luxCt/rbcL*^*wt *^and *rbcL-luxCt/rbcL*^- ^lines was digested with restriction enzymes and hybridized with the nucleotide probes according to the figure legend. Both *rbcL-luxCt/rbcL*^*wt *^and *rbcL-luxCt/rbcL*^- ^transformants show a *luxCt *hybridizing band at the expected size of 4.4-kb, while the WT strain shows no signal with the *luxCt *probe (Figure 3A, third panel from left). Additionally, only the *rbcL-luxCt/rbcL*^- ^transformant produced an *aphA6 *hybridizing band (2.7-kb; Figure 3A, second panel from left). Hybridization with the *Bam *HI-*Xho *I fragment from the p322 plasmid identifies a single 5.7-kb band in WT and a 3.4-kb band in the two transformants, and all bands are of the expected size (Figure 3A, left panel). Using an *rbcL *probe, a 4-kb band was detected in WT and the *rbcL-luxCt/rbcL*^*wt *^transformant, produced from the endogenous *rbcL *gene, while the *rbcL-luxCt/rbcL*^*wt *^transformant also contained a 3-kb *rbcL *hybridizing band from the *rbcL-luxCt *gene (Figure 3A, right panel). The *rbcL-luxCt/rbcL*^- ^strain contained the 3-kb *rbcL *band from the fusion protein and no endogenous *rbcL *band at 4-kb. These data demonstrate that the two transgenic lines are homoplasmic for the correctly integrated *rbcL-luxCt *and *aphA6 *transgenes, and the *rbcL-luxCt/rbcL*^- ^strain is lacking the endogenous *rbcL *coding region.

### Accumulation of *luxCt *mRNA in transgenic strains

To determine if the *luxCt *gene was transcribed in transgenic *C. reinhardtii *chloroplasts, Northern blot analysis of total RNA was used. Twenty micrograms of total RNA, isolated from WT and the two transgenic strains, was separated on denaturing agarose gels and blotted onto nylon membranes. Duplicate filters were stained with methylene blue (Figure 3B, left panel), or hybridized with a ^32^P-labeled luxCt probe (Figure 3B, middle panel), or rbcL probe (Figure 3B, right panel). The rbcL probe identified a 1.5-kb *rbcL *mRNA transcript produced from the endogenous *rbcL *gene in both the WT and in the *rbcL-luxCt/rbcL*^*wt *^transgenic strain, but not in the *rbcL *replacement strain *rbcL-luxCt/rbcL*^- ^(lower bands; Figure 3B, right panel). A larger transcript corresponding to the *rbcL-luxCt *chimeric mRNA was identified in the both the *rbcL-luxCt/rbcL*^*wt *^and *rbcL-luxCt/rbcL*^- ^strains (upper bands; Figure 3B, right panel), but not in WT. Hybridization of the filters with the luxCt probe identified the chimeric *rbcL-luxCt *mRNA of the predicted 4.3-kb in both the *rbcL-luxCt/rbcL*^*wt *^and *rbcL-luxCt/rbcL*^- ^lines, while no *luxCt *signal was observed in WT (Figure 3B, middle panel). These data confirm that the transgenic lines generated are producing the expected *rbcL *and *rbcL-luxCt *mRNAs.

### Accumulation and processing of the Rubisco LSU-LUXCt fusion protein yields individual Rubisco LSU and LUXCt proteins of the expected molecular weights

**Figure 4:**
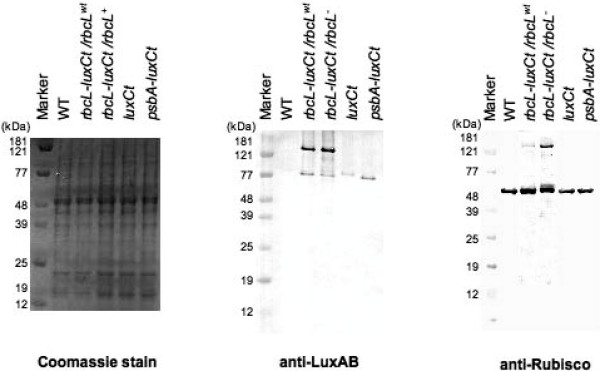
**Detection of LUXCt protein accumulation in transgenic *C. reinhardtii *strains**. Ten or two micrograms of total proteins from untransformed wild type (WT), *rbcL-luxCt *fusion transformant (*rbcL-luxCt/rbcL*^*wt*^), combined *rbcL-luxCt *fusion/*rbcL *knock-out transformant (*rbcL-luxCt/rbcL*^-^), and the *luxCt *expressing strains, *luxCt *and *psbA-luxCt*, were separated by 12% SDS-PAGE and stained with Coomassie blue (left panel), or blotted onto nitrocellulose membranes and decorated with anti-LUXAB (middle panel) or anti-Rubisco (right panel) antibodies. Protein levels from these strains were compared to a strain expressing LUXCt from the p322 integration site. Proteins were visualized on Western blots by alkaline phosphatase activity staining. Proteins were derived from cultures grown on TAP.

Western blot analysis of the different transgenic lines was used to analyze the accumulation of both processed and unprocessed Rubisco LSU and LUXCt proteins. Ten micrograms of total protein from WT, *luxCt*-expressing, and from the *rbcL-luxCt/rbcL*^*wt*^, and *rbcL-luxCt/rbcL*^- ^fusion strains was separated by SDS-PAGE and either stained with Coomassie blue or blotted onto nitrocellulose filters and hybridized with anti-Rubisco or anti-LUXAB antiserum. The Coomassie staining (Figure 4, left panel) indicated that equal amounts of protein (10 μg) were loaded in each lane, and that the transgenic lines accumulate a similar set of proteins as compared to WT. Western blot analysis of the same samples identified a 78-kDa band, corresponding to the cleaved LUXCt protein, in all of the *luxCt *transgenic lanes (Figure 4, middle panel). The anti-LUXAB antibody also identified a Rubisco LSU-LUXCt fusion protein (~133-kDa) in *rbcL-luxCt/rbcL*^*wt *^and *rbcL-luxCt/rbcL*^- ^transgenic strains. No signal was observed in the WT *C. reinhardtii *lane, as expected. The same samples were also assayed for Rubisco protein accumulation by Western blot analysis (Figure 4, right panel). A monomeric or cleaved Rubisco LSU protein (approximately 55-kDa) was identified in WT and all of the transgenic lines. Moreover, an additional Rubisco LSU-LUXCt fusion protein (133-kDa) was identified in *rbcL-luxCt/rbcL*^*wt *^and *rbcL-luxCt/rbcL*^- ^transgenic strains.

### Analysis of luciferase activity in transgenic *C. reinhardtii *chloroplasts

**Figure 5:**
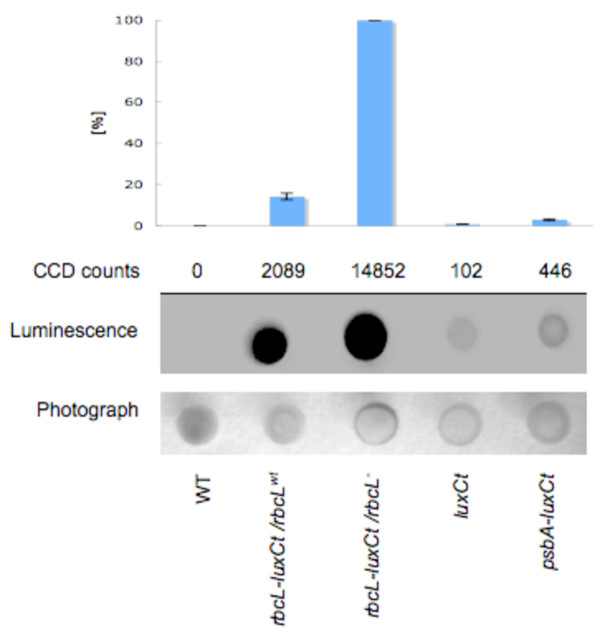
**Quantification of luciferase in *luxCt *transgenic lines**. Equal cell numbers of WT and *luxCt *transgenic lines (*luxCt*, *psbA-luxCt, rbcL-luxCt/rbcL*^*wt*^, and *rbcL-luxCt/rbcL*^-^) were spotted on solid Tris-acetate-phosphate (TAP) media. The samples were treated with a decanal and visualized on a CCD camera for luminescence (middle panel) or photographed under reflective light (bottom panel). The mean values (n = 4) of CCD counts were reported as total counts per second per sample. CCD counts were acquired and the highest value set to 100%. Relative values were calculated as a percentage of the highest value. The mean values (n = 4) ± SE were presented (top panel).

To determine whether recombinant protein expression differed between transgenic lines, luciferase activity was measured by luminescence assays using a CCD camera. For luciferase activity assays, 1 × 10^6 ^cells from WT and each of the transgenic lines were spotted onto solid Tris-acetate-phosphate (TAP) media. The plates were exposed to decanal, the substrate for LUXCt, and luciferase activity was measured using a CCD camera and Night Owl quantification software. As shown in Figure 5, the WT cells have no detectable luminescence signal, as expected. The LUXCt control strains, *luxCt *driven by the *rbcL *or *psbA *promoter and UTR (*rbcL-luxCt *and *psbA-luxCt *strains), show lower luciferase activity compared to LUXCt fusion strains at this cell density (Figure 5, top panel and CCD counts). Luciferase activity for the Rubisco LSU-LUXCt fusion protein is at least 4.7 times greater than for the LUXCt protein alone, and this increased luciferase activity is seen in both the *rbcL-luxCt/rbcL*^*wt *^and the *rbcL-luxCt/rbcL*^- ^strains (Figure 5, top panel and CCD counts). Although the *rbcL-luxCt/rbcL*^- ^strain produced the highest luminescence signal (Figure 5, middle panel), the transgenic lines appear similar to WT cells when visualized under reflective light (Figure 5, bottom panel).

## Discussion

This study provides the first example of genetically manipulating an endogenous chloroplast protein to increase recombinant protein accumulation in algae. By introducing an *rbcL*-*luxCt *fusion gene we could increase activity of a recombinant luciferase protein up to 140-fold compared to the luciferase gene expressed alone, and ~33 times better than the best previous promoter/UTR combination [7].

As recombinant proteins fused to endogenous proteins are generally of little or no industrial or clinical use, we chose a fusion strategy in which we engineered the desired recombinant protein (LUXCt) to be cleavable from an endogenous chloroplast protein (Rubsico LSU). To accomplish this, we incorporated the 32 amino acid N-terminal chloroplast transit peptide of the nuclear-encoded preFd protein, an amino acid sequence known to be proteolytically removed from preFd upon translocation of the protein into the chloroplast [22,29]. Using this strategy, we demonstrate the production of a full-length Rubisco LSU-LUXCt fusion protein capable of undergoing *in vivo *proteolytic processing to yield: 1) a biologically-active, mature LUXCt recombinant protein 2) a Rubisco LSU monomer able to incorporate into the multi-subunit Rubisco holoenzyme and support photoautotrophic growth.

We further show that by expressing the fusion construct in an *rbcL *knock-out background, LUXCt protein activity is greatly enhanced relative to its expression in an *rbcL *WT background. We suspect that by making the fusion protein the sole source of Rubisco LSU for incorporation into the Rubisco holoenzyme, the transgene is more highly expressed and thus LUXCt is more highly expressed. Taken together, our data suggest that the forced expression of a transgenic protein in the form of fusion to an endogenous protein required for photoautotrophic growth represents a novel strategy for the increased accumulation of a recombinant protein in the chloroplast of the unicellular green algae *C. reinhardtii*.

Although we observed *in vivo *processing of LUXCt from Rubisco LSU, the percent effective processing as judged by western blot analysis is quite different when comparing Rubisco LSU to LUXCt (Figure 4, far right panel). Examination of Figure 4 shows that Rubisco LSU is predominantly processed to the mature length, while examination of LUXCt accumulation shows that only about 20% is processed to the mature size. This suggests that processing of the protein yields a stable Rubisco LSU and an unstable LUXCt, which is then degraded. Although western blotting indicates that the *rbcL-luxCt/rbcL*^*wt *^and the *rbcL-luxCt/rbcL*^- ^strains each produce 3–5 times more LUXCt than the *psbA-luxCt *strain, LUXCt activity is ~33 times higher in the *rbcL-luxCt/rbcL*^- ^strain compared to the *psbA-luxCt *strain. This suggests that LUXCt must be much more active when expressed as a fusion protein in an endogenous *rbcL *knock-out background.

The world-wide demand for clinically and industrially-relevant recombinant proteins continues to grow, as does the requirement to produce such proteins in a cost-effective manner at ever-increasing amounts. Since the advent of recombinant DNA technology, several biological systems have been harnessed to produce recombinant proteins, including bacteria, yeast, mammalian and insect cell culture, and higher plants. While the success of large-scale production of recombinant proteins of medicinal value (e.g. insulin, antibodies, growth hormone) using bacterial and mammalian cell culture systems can not be refuted, such systems are not without their disadvantages. Bacterial expression is limited by the types of eukaryotic proteins that can be expressed at high levels while maintaining solubility, proper folding, and the correct post-translational modifications. Production of recombinant proteins in mammalian cell culture, most notably the Chinese hamster ovary [30] expression system, addresses many of the problems that plague bacterial systems. Although such mammalian cell culture systems are highly efficacious at producing properly-folded, correctly-modified eukaryotic proteins, they have several drawbacks: high initial and fixed operating costs, slow temporal progression from drug lead to large-scale production, susceptibility to viruses and contamination by fungi and bacteria. Clearly, there remains a significant need for a recombinant protein expression platform that combines the versatility of mammalian cell culture and the cost-effectiveness of bacterial systems with several other attributes lacking in both technologies.

Recombinant protein expression in the unicellular green algae *C. reinhardtii*, has now been demonstrated for several proteins of both prokaryotic and eukaryotic origin [3]. Proteins expressed in this alga have been shown to have biological activity equivalent to their native counterparts, demonstrating the efficacy of algae for producing functional exogenous proteins [7]. *C. reinhardtii *is a very attractive biological system for the large-scale production of medicinally and industrially-relevant proteins for several reasons. It is inexpensive to grow, free of invading viruses, has easily transformed nuclear and chloroplast genomes and can be grown photoautotrophically, eliminating the need for a carbon source in the growth medium, thereby limiting contamination of the algal culture.

The chloroplast is a unique environment for the production of recombinant proteins as the chloroplast contains a variety of chaperones [31,32] and protein disulfide isomerases [33] that allow for the correct folding of complex mammalian proteins, yet the chloroplast lacks the ability to perform several post-translational protein modifications in mammalian systems, such as glycosylation. However, there are a number of circumstances when post-translational modifications are not desirable, and chloroplasts appear to be an ideal system to make proteins of this type. Algal chloroplasts also appear to lack gene silencing mechanisms (e.g. RNAi, miRNA-mediated gene silencing) and transgenes introduced into the chloroplast genome appear to be quite stable [3]. Although *C. reinhardtii *has many attributes that make it both suitable for recombinant protein expression and superior to current methodologies for the production of certain proteins, achieving routinely robust transgene expression in the algal chloroplast is not commonplace.

Genetic engineering of the *C. reinhardtii *chloroplast represents a promising strategy to address the issues of cost, scalability and adaptability faced by current expression methodologies, but only if robust recombinant protein expression can be achieved. Several strategies have been employed, by our laboratory and others, to increase the accumulation of recombinant proteins in *C. reinhardtii *chloroplasts. Codon optimization resulted in an up to 80-fold increase in recombinant protein accumulation [14,15,34], while a combinatorial analysis of a variety of endogenous promoters and untranslated regions identified a variety of expression levels, some up to ten times better than others [16]. Finally, replacing the endogenous *psbA *gene with a chimeric gene containing the *psbA *promoter and 5' UTR fused to a mammalian coding region result in very high levels of expression of that particular mammalian protein, approaching 10% of the TSP [7]; however, other recombinant proteins expressed at much lower levels using the exact same chimeric promoter/5' UTR. Highly-variable recombinant protein expression is found in all expression systems, including bacterial and mammalian cell culture. Some proteins with problematic expression characteristics in bacterial systems show a dramatic increase in accumulation when the recombinant protein is expressed as a fusion with a protein previously shown to express at high levels in the bacterial system, and hence we followed this strategy with the abundant endogenous Rubisco LSU protein in chloroplasts.

Analysis of transcription rates of chimeric constructs using a beta-glucuronidase (GUS) reporter driven by various endogenous 5' promoter/UTR elements revealed a possible interdependence between the *rbcL *5' leader region and the first 257 bp of the *rbcL *coding sequence [17]. In our previous studies, we constructed chimeric mRNAs utilizing the 5' UTR/promoter and 3' UTR elements derived from several endogenous chloroplast genes (e.g. *rbcL*, *atpA*, *psbA*, *psbD*) to drive the expression of different exogenous coding regions including GFP, LUXCt and bovine mammary serum amyloid albumin (M-SAA) [7,14-16]. For each transgene studied, we achieved good accumulation of the transgenic mRNA, often mirroring the steady-state levels of the endogenous transcript, but recombinant protein accumulated to much lower levels than did the endogenous proteins regulated by the same promoters and UTRs. Indeed, there is evidence to suggest that transcript abundance is not limiting for protein synthesis of *C. reinhardtii *chloroplast-encoded genes [35].

## Conclusion

These results demonstrate the utility of fusion proteins in the *C. reinhardtii *chloroplast as a means to increase accumulation of recombinant proteins that are difficult to express. It will be interesting to more rigorously test our expression system by fusing other hard-to-express proteins downstream of *rbcL*, express them in the *rbcL *knock-out background and determine how broad the applicability of our system is. It is possible that our expression strategy will succeed beyond LUXCt production and that a wide variety of recombinant proteins might be expressed at high levels using this cleavable fusion strategy. While this awaits further experimentation, the results of our work are encouraging and suggest that expressing recombinant proteins in the form of cleavable fusions to highly-expressed, endogenous chloroplast proteins might be a robust platform by which recombinant proteins can be produced in algal chloroplasts.

## Methods

### *Chlamydomonas reinhardtii *strains, transformation, and growth conditions

Transformations were carried out on *C. reinhardtii *strain wild type (WT) 137c (mt+). Cells were grown to late log phase (approximately 7 days) in the presence of 40 mM 5-fluorodeoxyuridine in liquid TAP medium [36] at 23°C under constant illumination of 3000 lux on a rotary shaker set at 100 r.p.m. Fifty ml of cells was harvested by centrifugation at 4000 g at 4°C for 5 min. The supernatant was decanted, and cells were resuspended in TAP medium at 0.5 × 10^8 ^cells/ml for subsequent chloroplast transformation by particle bombardment, as described previously by Cohen *et al*. [8]. All transformations were carried out under spectinomycin selection (150 μg ml^-1^) in which resistance was conferred by co-transformation with plasmid p228 carrying a mutant allele of the 16S rRNA gene which confers resistance to spectinomycin [13,28]. A selectable marker gene *aphA6*, which confers resistance to kanamycin, was used for the additional transformation [12].

### Construction of *C. reinhardtii *chloroplast vectors

All DNA and RNA manipulations were carried out essentially as described previously [8,37]. The chloroplast codon-optimized coding regions of the *luxCt *gene as well as for the other recombinant proteins were synthesized *de novo *according to the previous methods from a pool of primers, each 40 nucleotides in length [14,22,23]. The 5'- and 3'-terminal primers used in this assembly contained restriction sites for *Nde*I and *Xba*I, respectively. The *rbcL *promoter and 5' UTR and the *rbcL *3' UTR fragments were generated as described previously [15]. The vector constructed previously, eliminated photosynthesis by *psbA *gene replacement [7], and was used as an internal standard for endogenous *psbA *gene deletion (Figure 1A). The chloroplast transformation plasmid p322 was constructed as described by Franklin *et al*. [15].

### Southern and Northern blotting

Southern and Northern blotting procedures, as well as ^32^P-dCTP labeling methods in the generation of DNA probes were carried out as described previously [8,37]. Radioactive probes used on Southern blots included the 2-kb *luxCt *coding region (lux probe as in Figure 1A), the 1.4-kb fragment from the *rbcL *cDNA coding region (rbcL probe as in Figure 1A), and the 2.0-kb *Bam*HI-*Xho*I p322 fragment (Bam-Xho probe as in Figure 1A). A 0.8-kb fragment from the *aphA6 *coding region was also used to detect *aphA6 *insertion (aphA6 probe as in Figure 1A). The lux and rbcL probes were used to detect *luxCt *and *rbcL *mRNA on Northern blots.

### Recombinant protein expression, Western blotting, and luminescence assays

For whole-cell luminescence assays, *C. reinhardtii *cultures were grown on solid TAP media under constant illumination (1000 lux) and the cells were resuspended in TAP medium at 1 × 10^6 ^cells/10 μl for *rbcL-luxCt/rbcL*^*wt*^, and *rbcL-luxCt/rbcL*^- ^strains. A WT negative control strain and two positive control strains *luxCt *(*luxCt *driven by *rbcL *promoter) and *psbA-luxCt *(the *psbA *promoter driving *luxCt *expression replacing the endogenous *psbA *locus) were included. The cells were placed on solid TAP medium, decanal was swabbed onto the plate lid, and the cells were incubated for 5 min prior to visualization on a CCD camera. The plate was visualized by luminescence imaging using the Night Owl CCD camera, and total luminescence was reported as counts per second per spot.

WT and recombinant strains of approximately equal cell number were grown in TAP medium [36] at 23°C under constant illumination of 1000 lux on a rotary shaker set at 100 r.p.m. As a positive control, protein from a LUXCt expressing strain [7], *luxCt*, was immunoblotted for LUXCt and Rubisco expression. For Western blot analysis, proteins were isolated from *C. reinhardtii *utilizing a buffer containing 750 mM Tris-HCl (pH 8.0), 15% sucrose (w/v), and 100 mM β-mercaptoethanol. Total proteins from *C. reinhardtii *were used in Western blot analysis. Western blotting procedures were carried out as described previously [8] using rabbit anti-LUXAB (gift of Susan Golden) and anti-Rubisco primary antibodies, followed by detection with an alkaline phosphatase-conjugated goat anti-rabbit secondary antibody (Sigma, St Louis, MO, USA).

## Authors' contributions

MM carried out all of the experiments and analyses with the exception of generating the *rbcL-luxCt/rbcL*^*wt *^transformant, participated in the study design, wrote and edited the manuscript. REH conceived the study, designed and constructed the plasmid p322-*rbcL-luxCt*, and contributed to the writing of the manuscript. SPM contributed to the writing of the manuscript and to the experimental design. All authors read and approved the final manuscript. MM and REH contributed equally to this work.
